# Mechanism of RecQ helicase mechanoenzymatic coupling reveals that the DNA interactions of the ADP-bound enzyme control translocation run terminations

**DOI:** 10.1093/nar/gku1333

**Published:** 2014-12-24

**Authors:** Kata Sarlós, Máté Gyimesi, Zoltán Kele, Mihály Kovács

**Affiliations:** 1 Department of Biochemistry, ELTE-MTA ‘Momentum’ Motor Enzymology Research Group, Eötvös University, Pázmány P. s. 1/c, Budapest, H-1117, Hungary; 2 Department of Medical Chemistry, University of Szeged, Dóm sqr. 8. Szeged, H-6720, Hungary

## Abstract

The processing of various DNA structures by RecQ helicases is crucial for genome maintenance in both bacteria and eukaryotes. RecQ helicases perform active destabilization of DNA duplexes, based on tight coupling of their ATPase activity to moderately processive translocation along DNA strands. Here, we determined the ATPase kinetic mechanism of *E. coli* RecQ helicase to reveal how mechanoenzymatic coupling is achieved. We found that the interaction of RecQ with DNA results in a drastic acceleration of the rate-limiting ATP cleavage step, which occurs productively due to subsequent rapid phosphate release. ADP release is not rate-limiting and ADP-bound RecQ molecules make up a small fraction during single-stranded DNA translocation. However, the relatively rapid release of the ADP-bound enzyme from DNA causes the majority of translocation run terminations (i.e. detachment from the DNA track). Thus, the DNA interactions of ADP-bound RecQ helicase, probably dependent on DNA structure, will mainly determine translocation processivity and may control the outcome of DNA processing. Comparison with human Bloom's syndrome (BLM) helicase reveals that similar macroscopic parameters are achieved by markedly different underlying mechanisms of RecQ homologs, suggesting diversity in enzymatic tuning.

## INTRODUCTION

Transmission and maintenance of genetic information requires the action of helicases, enzymes that catalyze nucleic acid (NA) strand separation and restructuring driven by nucleoside triphosphate (NTP) hydrolysis (1). RecQ-family enzymes, belonging to helicase superfamily (SF) 2, are conserved from bacteria to humans (2). Their key roles in homologous recombination (HR)-based DNA repair are indicated by severe cancer-causing hereditary syndromes resulting from their deficiency (2,3). *Escherichia coli* expresses a single RecQ homolog, with key roles in the suppression of illegitimate recombination (4), repair of DNA double-stranded breaks (5,6) and stabilization of stalled replication forks (7,8).

DNA-restructuring activities of RecQ (and many other) helicases are based on mechanoenzymatic coupling of their adenosine triphosphate (ATP)-hydrolytic activity to unidirectional movement (translocation) along single-stranded (ss)DNA. Previous studies showed that *E. coli* RecQ helicase effectively couples its ATP-hydrolytic activity to stepping cycles along ssDNA, and stepping occurs with a moderate processivity (36–310 nt/run) (9,10). Mechanistic understanding of the coupling mechanism requires knowledge of the kinetics of substeps of the adenosine triphosphatase (ATPase) enzymatic cycle (ATP binding, hydrolysis, product release) and the coupling of these processes to the DNA interaction of the helicase (11–19). To this end, we elucidated the ATPase kinetic mechanism of DNA-free and ssDNA-bound *E. coli* RecQ using rapid transient and steady-state kinetic analysis. We found that the marked (about 100-fold) activation of the RecQ ATPase by ssDNA is practically entirely due to the kinetic enhancement of the chemical step of ATP cleavage, which is rate-limiting in all conditions. ATP hydrolysis occurs productively due to rapid release of nascent inorganic phosphate (P_i_). Synthesis of current results with earlier data on the RecQ-ssDNA interaction (11) allowed the construction of a mechanoenzymatic model of the translocation-coupled ATP hydrolytic cycle. The model reveals that the ADP-bound enzymatic state is populated by a small fraction of RecQ molecules during ssDNA translocation, but it is responsible for the majority of translocation run terminations (DNA detachment events) due to its relatively rapid dissociation from ssDNA. The DNA interactions of this state, which may strongly depend on the structure of the DNA substrate being processed, may therefore control the processivity and outcome of DNA processing events. Comparison of *E. coli* RecQ and human Bloom's syndrome (BLM) ATPase cycles reveals marked differences between DNA activation mechanisms of RecQ homologs that probably contribute to their functional diversity.

## MATERIALS AND METHODS

### Reagents

Unless otherwise stated, all materials were from Sigma-Aldrich. P_i_ standard was from Merck. ATP was from Roche Applied Science. mdATP (3′-(N-methylanthraniloyl)-2′-deoxy-ATP) was from Jena Bioscience. γ-^32^P-ATP was from Institute of Isotopes Co. Ltd. (Hungary). mdADP was prepared from mdATP by mixing 0.5-μM rabbit skeletal muscle myosin subfragment-1 with 1-mM mdATP in SF50 buffer (50-mM Tris-HCl pH 7.5, 50-mM NaCl, 1-mM dithiothreitol (DTT), 5-mM MgCl_2_) and incubating at 25°C overnight to achieve complete hydrolysis to mdADP. (This solution was diluted at least 15 times for the experiments.) A 54-mer deoxythymidine oligonucleotide (dT_54_, obtained from VBC-Biotech) was used as ssDNA ligand. ϵ_260_ = 8400 M^−1^cm^−1^nt^−1^ was used for dT_54_ concentration determination. DNA concentrations are expressed as those of dT_54_ molecules (as opposed to constituent nt units). RecQ was expressed and purified as in (9).

### Kinetic experiments

Unless otherwise stated, all measurements were carried out in SF50 buffer at 25°C. Stopped-flow experiments were carried out in KinTek SF-2004 and BioLogic SFM 300 apparatuses. Quenched-flow experiments were performed in a KinTek RQF-3 instrument. Post-mixing concentrations are stated in all experiments. In experiments requiring nucleotide-free RecQ, nucleotide contamination was removed by pre-incubation with 0.02-U/ml apyrase for 15 min at 25°C. P_i_ liberation from ATP was followed using a fluorescently labeled P_i_ binding protein (MDCC-PBP) (12). MDCC-PBP calibration was performed as described earlier (13,14). mdATP and mdADP were excited at 280 nm and fluorescence emission was detected through a 420-nm long-pass filter utilizing FRET (Förster Resonance Energy Transfer) from aromatic residues of RecQ.

Steady-state ATPase measurements were carried out in SF50 buffer plus 50-μg/ml bovine serum albumin using a pyruvate kinase-lactate dehydrogenase (PK-LDH) linked assay (14-U/ml PK, 20-U/ml LDH, 1-mM ATP, 1-mM phosphoenol pyruvate, 200-μM NADH (nicotinamide adenine dinucleotide, reduced form)). Time courses of NADH absorbance (*ϵ*_340 nm_ = 6220 M^−1^cm^−1^) were followed in a Shimadzu UV-2101PC spectrophotometer.

### Oxygen exchange experiments

Samples were prepared by incubating 1-μM RecQ and 1-mM ATP in SF50 buffer containing 40 ± 2% ^18^O water for 4 h in the absence or for 30 minutes in the presence of 2 μM dT_54_, to achieve complete ATP hydrolysis. The ^18^O content of water was determined by measuring ^18^O incorporation into P_i_ upon hydrolysis of PCl_5_. Samples were then mixed with an equal volume of 10% activated charcoal in distilled water and centrifuged at 14 krpm in a tabletop microcentrifuge for 1 min at 25°C to remove protein and residual nucleotides. The supernatant was loaded on a DOWEX column pre-equilibrated with distilled water. P_i_ was eluted with 10-mM HCl and lyophilized to dryness. Samples were then analyzed by mass spectrometry (MS).

MS measurements were performed on a Waters/Micromass Q-TOF Premier mass spectrometer (Waters/Micromass, Manchester, UK) equipped with a microelectrospray ionization source. Samples were dissolved in MilliQ water and 2-μl aliquots were diluted 20 times with methanol. Diluted samples were filled into gold-coated borosilicate glass nanospray capillary tips. During the measurement the spray voltage was set to 1 kV. The instrument was operated in negative ion mode observing differentially ^18^O-exchanged H_2_PO_4_^−^ ions at *m*/*z* ratios of 97, 99, 101 and 103. Molar ratios of different H_2_PO_4_^−^ isotopic species were calculated based on their peak intensities.

### Data analysis

Data analysis, fitting and simulations were performed using OriginLab 8.0 (Microcal Corp.), KinTek SF-2004 and KinTek Global Kinetic Explorer software. Reported values are means ± SEM of three to six rounds of experiment.

## RESULTS

### Rapid and reversible nucleotide binding to RecQ is largely unaffected by DNA

**Figure 1.**
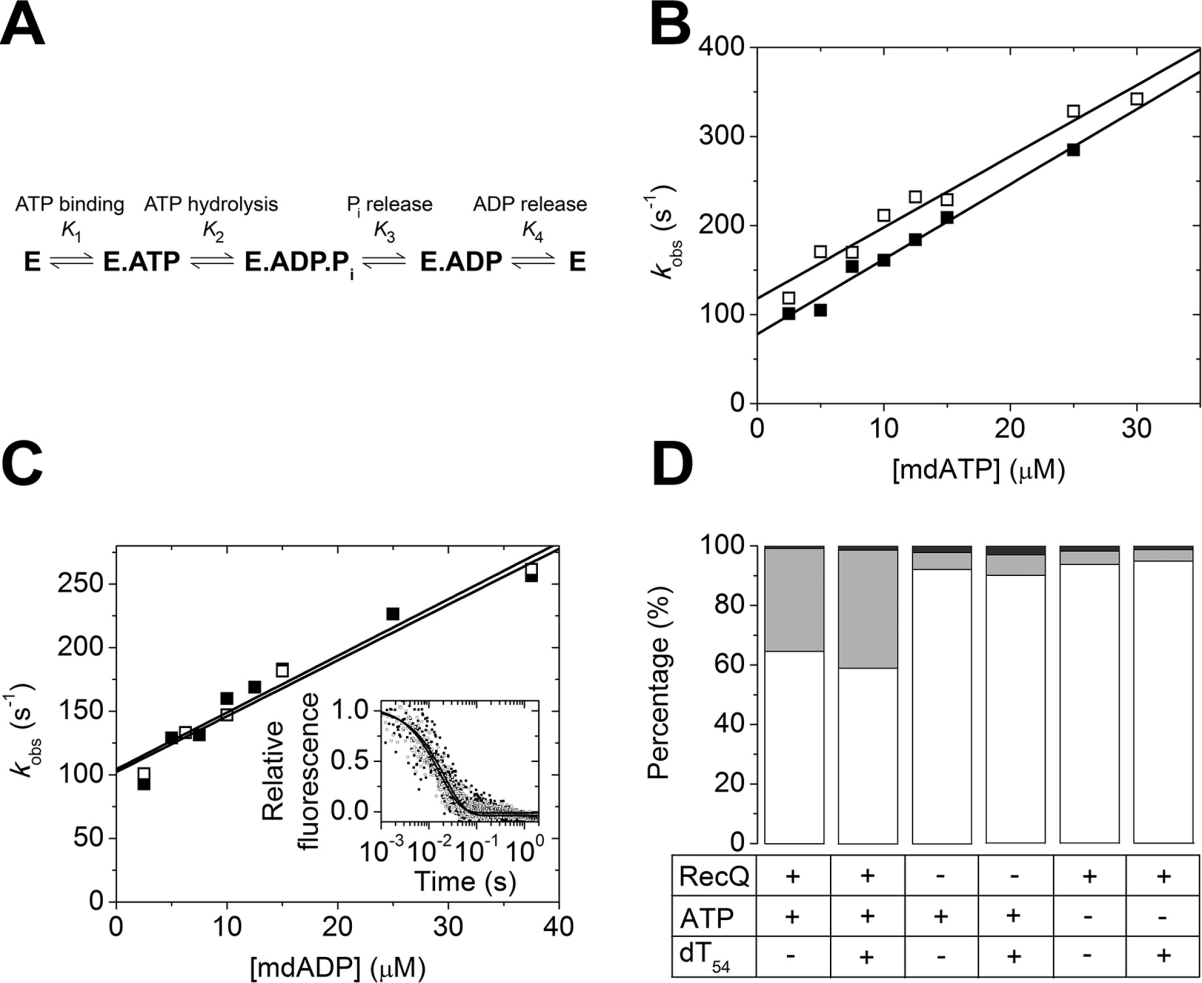
RecQ helicase exhibits rapid and reversible nucleotide binding and productive ATP hydrolysis. (**A**) Kinetic model of the RecQ ATPase cycle. ‘RecQ’ denotes either the DNA-free or DNA-bound enzyme, driving the basal or DNA-activated ATPase cycles, respectively (cf. Table 1). P_i_, inorganic phosphate. (**B,C**) Observed rate constants (*k*_obs_) of mdATP (B) and mdADP (C) binding, obtained from single-exponential fits to stopped-flow transients recorded upon rapidly mixing 1-μM RecQ with increasing concentrations of md-nucleotides in the presence (solid symbols) and absence (open symbols) of 1-μM dT_54_. Linear fits (lines) yielded parameters (*k*_1_, *k*_-1_ for (B); *k*_-4_, *k*_4_ for (C)) listed in Table 1. Inset in (C): normalized mdADP ‘chasing’ transients upon rapidly mixing 1-μM RecQ plus 20-μM mdADP with 0.5-mM unlabeled ATP in the presence (gray) and absence (black) of 2-μM dT_54_ (pre-mixed with RecQ). Single-exponential fits to the transients resulted in similar *k*_4_ values as did the binding experiments (Table 1). (**D**) Distribution of H_2_PO_4_^−^ isotopic species resulting from complete hydrolysis of 1*-*mM ATP in 40% ^18^O-containing water by 1-μM RecQ in the absence and presence of 2-μM dT_54_, alongside control experiments as indicated. White bars: no ^18^O; light gray bars: one ^18^O; gray bars: two ^18^O atoms incorporated. Mass spectrometric analysis.

We monitored nucleotide binding (steps 1 and 4 in Figure 1A) to RecQ helicase in stopped-flow experiments by rapidly mixing the enzyme with excess amounts of fluorescently labeled 3′-(N-methylanthraniloyl)-2′-deoxy (md) nucleotides (mdATP, mdADP) in the presence and absence of dT_54_ ssDNA ligand (Figure 1B). dT_54_ was premixed with RecQ at saturating concentration, as determined in our earlier experiments (9,11). The time courses of the observed md-nucleotide fluorescence increase were fitted by single-exponential functions. The observed rate constants (*k*_obs_) of the pseudo-first-order reactions showed linear dependence on nucleotide concentration (Figure 1B and C). The slopes of the plots, reflecting nucleotide binding rate constants (*k*_1_ and *k*_–4_ for ATP and ADP, respectively; Figure 1A), were similar in the absence and presence of DNA (Figure 1B and C and Table 1). The *y* intercepts of the linear fits represent the sum of the rate constants of possible breakdown pathways of the nucleotide-bound enzyme intermediates (RecQ.ATP and RecQ.ADP, respectively). In the case of mdADP, this intercept purely represents the *k*_4_ ADP dissociation rate constant (Figure 1C and Table 1). In mdATP, the intercept represents the sum of the *k*_–1_ (ATP dissociation) and *k*_2_ (ATP hydrolysis) rate constants (Figure 1A and B and Table 1; see also below). The determined parameters were characteristic of rapid and reversible ATP and ADP binding by RecQ, which was largely independent of the presence of DNA (Table 1).

**Table 1.** Mechanoenzymatic parameters of the RecQ ATPase cycle

Parameter	Method of determination	DNA-free RecQ	ssDNA (dT_54_)-bound RecQ
*k*_1_ (μM^−1^s^−1^)	mdATP	8.0 ± 0.4	8.4 ± 0.5
*k*_–1_ + *k*_2_ (s^−1^)	mdATP	118 ± 8	78 ± 7
1/*K*_1_ (μM)	mdATP (*k*_–1_/*k*_1_)	15 ± 2	4.3 ± 0.8
	Quenched-flow ^a^	120 ± 30	60 ± 15
*k*_2_ (s^−1^)	Quenched-flow ^a^	0.15 ± 0.05	42 ± 2
*k_–_*_2_ (s^−1^)	Quenched-flow ^a^	>0.06	>100
(*k*_3_+*k*_–2_)*/k*_–2_	^18^O exchange	17 ± 3	11 ± 2
*k*_3_ (s^−1^)	Quenched-flow ^a^	>1	>1100
*k_–_*_3_ (s^−1^)	Quenched-flow ^a^	<1	<1
*k*_4_ (s^−1^)	mdADP binding	100 ± 10	100 ± 10
	mdADP chasing	67 ± 5	76 ± 10
	Quenched-flow ^a^	>200	>200
*k*_–4_ (μM^−1^s^−1^)	mdADP binding	4.5 ± 0.5	4.4 ± 0.4

Steady-state kinetics			
*k*_cat_ (s^−1^)	MDCC-PBP	0.53 ± 0.05	30 ± 3
	PK-LDH assay (+ P_i_) ^b^	0.40 ± 0.04 (0.46 ± 0.05)	32 ± 3 (26 ± 3)
	PK-LDH assay ^c^	0.13 ± 0.01	37 ± 1
*K*_ATP_ (μM)	MDCC-PBP	28 ± 2	16 ± 1
	PK-LDH assay ^c^	50 ± 10	20 ± 2
*n* (Hill coefficient)	MDCC-PBP	1.3 ± 0.1	1.1 ± 0.1
	PK-LDH assay ^c^	1.2 ± 0.2	1.0 ± 0.1

^a^Parameters determined in global fits constrained by experimentally determined *k*_1_, *k*_3_/*k*_–2_ and *k*_–4_ values.

^b^Determined at 1*-*mM ATP in the absence and presence of 100-nM dT_54_, at 200-nM and 15-nM RecQ, respectively. Values in parentheses were determined upon addition of 20-mM P_i_.

^c^Ref. (9).

The kinetics of dissociation of mdADP from RecQ (*k*_4_) was also measured independently in ‘chasing’ experiments. Upon rapid mixing of the RecQ.mdADP complex (with or without dT_54_ added) with excess unlabeled ATP, single-exponential transients were recorded (Figure 1C, inset). The *k*_obs_ values were in agreement with the fitted intercepts of binding experiments (Figure 1C and Table 1).

### RecQ-catalyzed ATP hydrolysis is productive due to rapid release of phosphate

We assessed the reversibility of the ATP hydrolysis step and the coupled release of P_i_ (inorganic phosphate) (steps 2–3 in Figure 1A) by ^18^O exchange measurements (Figure 1D). The principle of this method is that, during ATP hydrolysis, the oxygen atom of the attacking water becomes incorporated in the detaching γ-phosphate. If hydrolysis is performed in ^18^O-labeled water, oxygen incorporation can be monitored by MS. If ATP hydrolysis occurs irreversibly (*k*_3_ >> *k*_–2_), a P_i_ species with a single ^18^O atom will be produced. In the case of reversible hydrolysis, P_i_ species with two or more^18^O atoms will appear.

Upon the complete hydrolysis of 1-mM ATP by 1-μM RecQ in 40 ± 2% ^18^O-labeled water, we detected 35 ± 2% single ^18^O-labeled and 0.8 ± 0.1% double ^18^O-labeled P_i_ in the absence of DNA (Figure 1D). The ratio of the amounts of the two species reflects the probability of hydrolysis reversal (*k*_–2_/(*k*_3_+*k*_–2_)) as *L*_2_/*L*_1_ = *f* * *k*_–2_/(*k*_3_+*k*_–2_), where *L*_2_ and *L*_1_ are the amounts of double- and single-^18^O-labeled P_i_, respectively, *f* is the fractional ^18^O-content of water, *k*_–2_ is the rate constant of hydrolysis reversal and *k*_3_ is the rate constant of P_i_ release (Figure 1A). The data showed that P_i_ release is kinetically favored over hydrolysis reversal ((*k*_3_+*k*_–2_)/*k*_–2_ = 17 ± 3), reflecting productive hydrolysis of enzyme-bound ATP (Table 1). The situation was similar in the presence of dT_54_ where we detected 40 ± 3% single ^18^O-labeled and 1.4 ± 0.1% double ^18^O-labeled P_i_, defining (*k*_3_+*k*_–2_)/*k*_–2_ = 11 ± 2 (Figure 1D and Table 1). Based on these results, the amount of triple ^18^O-labeled P_i_ is expected to be less than 0.05% in all cases, which is below the detection limit. In control experiments performed in the absence of enzyme and/or ATP, 5–10% of the low P_i_ content of the samples was ^18^O-labeled, and the ratio of the amount of single and double ^18^O-labeled P_i_ was between 2 and 3 (carrying contributions from spontaneous hydrolysis and/or endogenous P_i_ contamination) (Figure 1D).

### The ATP hydrolysis step is rate-limiting and is markedly accelerated by DNA

**Figure 2.**
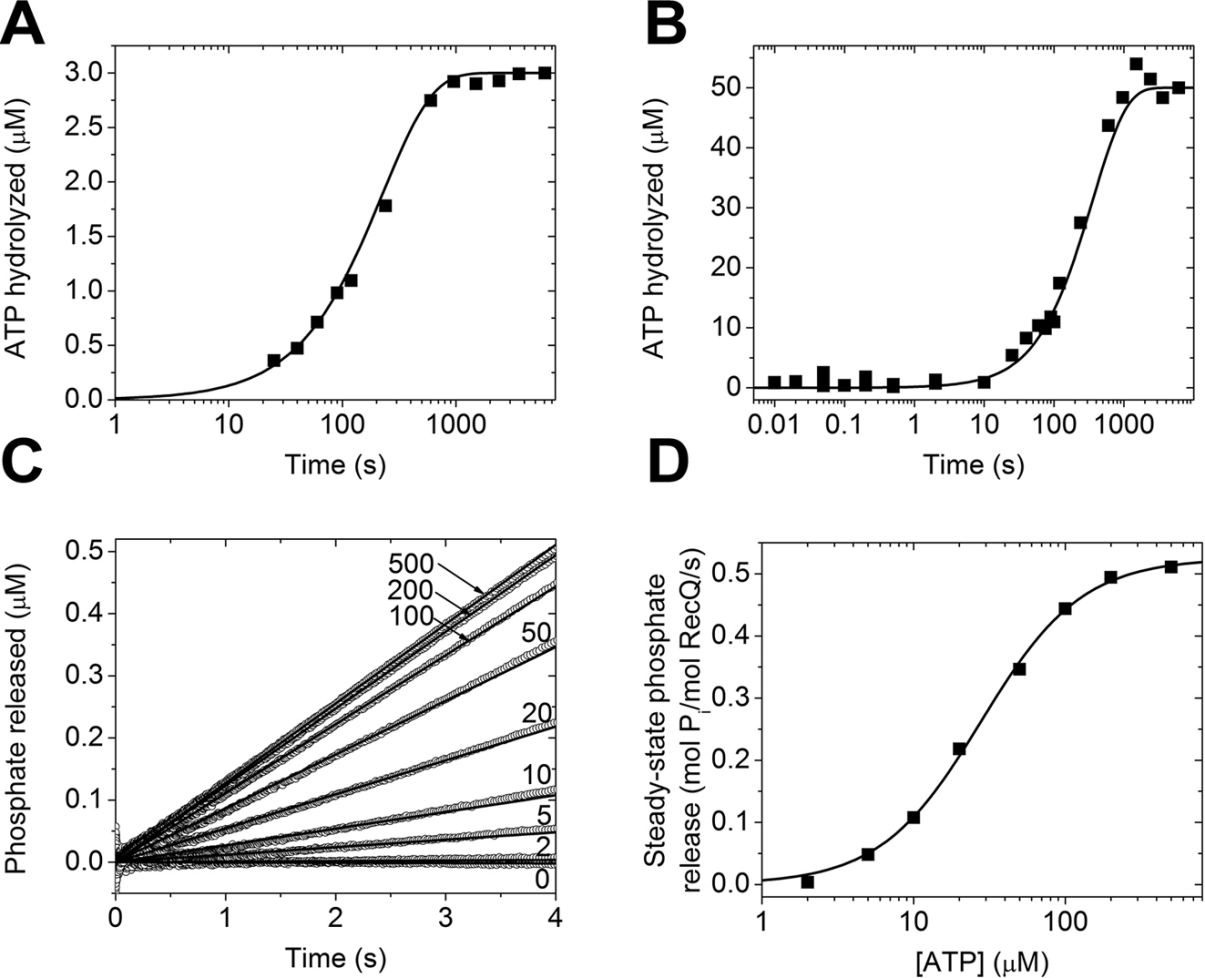
ATP hydrolysis is rate-limiting in the absence of DNA. Single (**A**) and multiple (**B**) turnover quenched-flow time courses of ATP hydrolysis, recorded upon mixing 4*-*μM RecQ with 3-μM (A) or 50-μM (B) γ-^32^P-ATP. Lines show global best-fits based on the model of Figure 1A. (**C**) Stopped-flow time courses of P_i_ release recorded upon rapidly mixing 0.25-μM RecQ with different ATP concentrations (indicated in μM). P_i_ production was monitored using MDCC-PBP fluorescence (2 μM in both syringes). (**D**) Steady-state ATPase (P_i_ production) activities determined from the slopes of linear fits (lines in (C)). Line shows best-fit using the Hill equation. Determined parameters are listed in Table 1.

We monitored the transient kinetics of ATP hydrolysis (step 2 in Figure 1A) in quenched-flow experiments by rapidly mixing 4-μM RecQ with 3-μM or 50-μM radiolabeled γ-^32^P-ATP to ensure single- and multiple-turnover conditions, respectively (Figure 2A and B). (In this context, ‘single turnover’ refers to a single ATP hydrolytic cycle, as opposed to single-run conditions referring to a sequence of ATP-driven translocation/unwinding steps upon a single encounter with the DNA track; see also below.) Time courses of ATP cleavage showed no observable burst phase, suggesting that the hydrolysis step is rate-limiting in the absence of DNA. To determine the rate constant of the hydrolysis step (*k*_2_), we performed global fitting analysis of the quenched-flow data, constrained by *k*_1_, *k*_–4_ and *k*_3_/*k*_–2_ values determined in other experiments (Figure 1). In addition to *k*_2_, nucleotide dissociation and P_i_ release/rebinding rate constants (*k*_–1_, *k*_4_, *k*_3_, *k*_–3_) were floated to obtain the best global fit. The best-fit *k*_2_ value was close to the steady-state ATPase *k*_cat_ ((9); see also below), confirming that ATP hydrolysis is rate-limiting in the absence of DNA (Table 1).

We recorded stopped-flow transient profiles of P_i_ release (step 3 in Figure 1) using a fluorescently labeled P_i_ binding protein (MDCC-PBP) (12). Upon rapidly mixing RecQ with excess ATP, no burst was observed before the onset of the linear steady-state phase of the reaction (Figure 2C). This behavior is expected as the rate-limiting ATP hydrolysis step precedes P_i_ release. Steady-state ATPase parameters, deduced from the ATP concentration dependence of the slope of P_i_ release records, were in line with those determined earlier in PK-LDH assays (Figure 2D and Table 1) (9).

**Figure 3.**
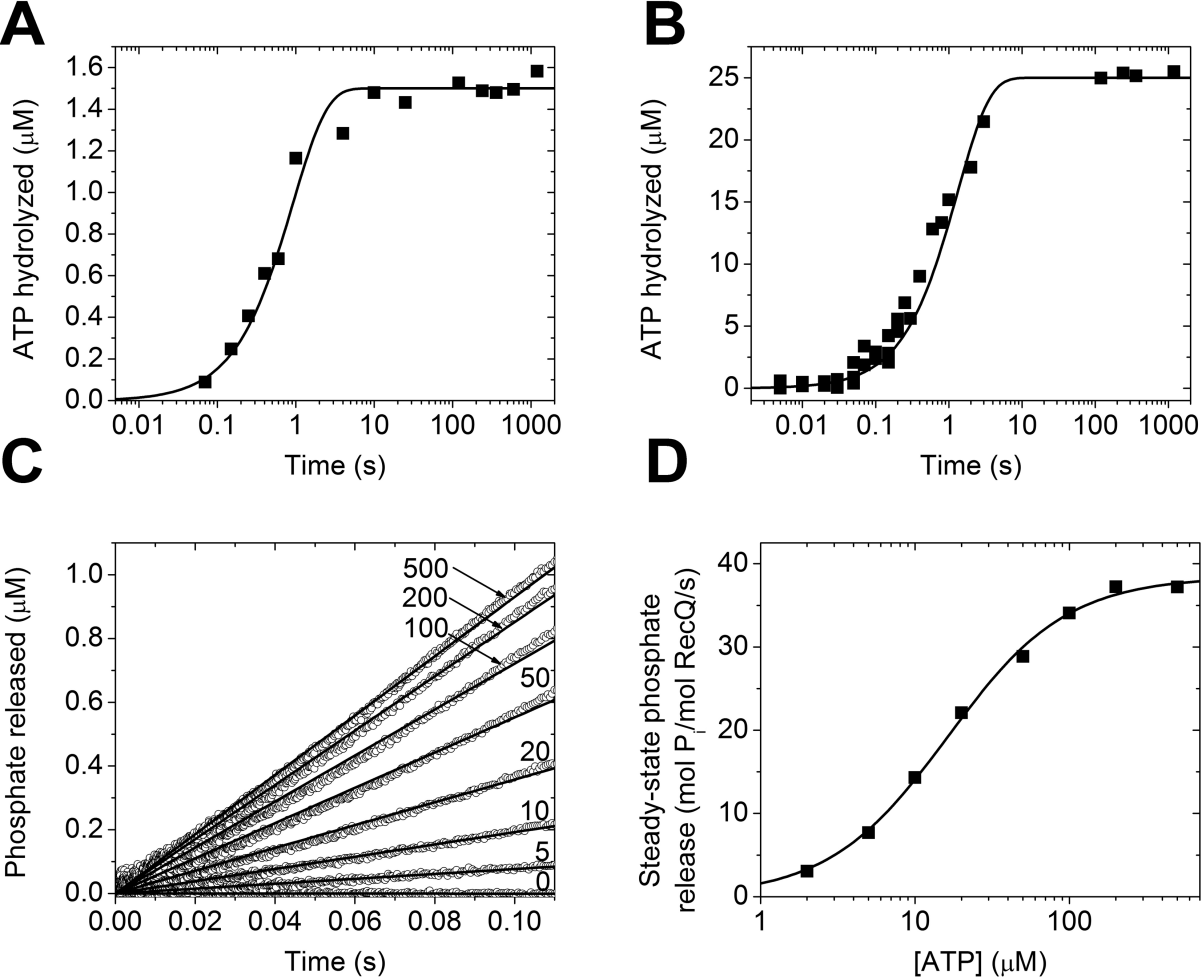
Rate-limiting ATP hydrolysis is markedly accelerated by DNA. Single (**A**) and multiple (**B**) turnover quenched-flow time courses of ATP hydrolysis, recorded upon mixing 2-μM RecQ plus 3-μM dT_54_ with 1.5-μM (A) or 25-μM (B) γ-^32^P-ATP. Lines show global best-fits based on the model of Figure 1A. (**C**) Stopped-flow time courses of P_i_ release recorded as in Figure 2C but in the presence of 0.5*-*μM dT_54_ (pre-mixed with RecQ). (**D**) Steady-state ATPase (P_i_ production) activities determined from the slopes of linear fits (lines in (C)). Line shows best-fit using the Hill equation. Determined parameters are listed in Table 1.

Similar to the situation in the absence of DNA, single- and multiple-turnover quenched-flow records obtained in the presence of dT_54_ showed no burst phase, suggesting that ATP hydrolysis remains rate-limiting even in the DNA-bound RecQ ATPase cycle (Figure 3A and B). However, ATP hydrolysis was markedly accelerated by DNA: global fits to the data (performed as described above) yielded an ∼100 times greater *k*_2_ value than in the absence of DNA (Table 1). As expected, P_i_ release (MDCC-PBP) stopped-flow transients did not show a burst phase even in the presence of DNA, in line with rate-limiting ATP hydrolysis (Figure 3C). Steady-state parameters of the DNA-activated RecQ ATPase activity, deduced from P_i_ release data, were in accordance with earlier PK-LDH results (Figure 3D and Table 1) (9). As found earlier in PK-LDH experiments (9), Hill coefficients deduced from the MDCC-PBP data were close to 1 both in the absence and presence of DNA, indicating that RecQ active sites cycle independently (Figures 2D and 3D and Table 1).

In the MDCC-PBP stopped-flow experiments, P_i_ release occurred irreversibly due to quasi-irreversible binding of P_i_ to MDCC-PBP. To assess the genuine irreversibility of P_i_ release from RecQ, we measured the effect of the addition of 20-mM P_i_ on the steady-state ATPase activity using a PK-LDH-linked assay. Both the DNA-free and DNA-bound ATPase activities of RecQ were largely unaffected by P_i_, indicating that the effective P_i_ dissociation constant (*K*_Pi,eff_ = *K*_3_*K*_2_/(*K*_2_+1)) is above 20 mM both in the absence and presence of DNA (Table 1).

### Mechanoenzymatic model identifies the RecQ.ADP state as the major source of run terminations controlling translocation processivity

**Figure 4.**
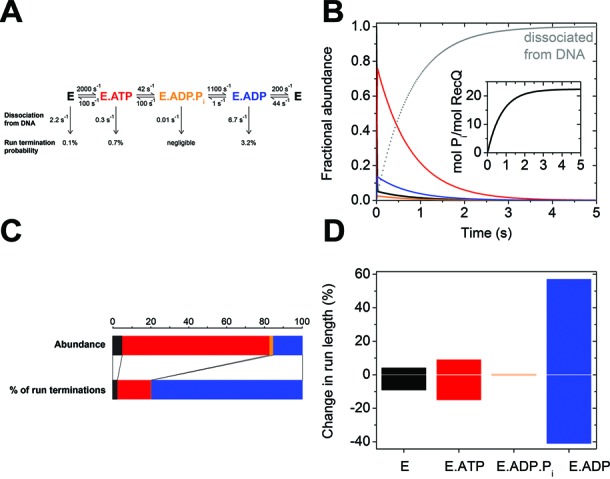
Mechanoenzymatic model identifies the RecQ.ADP state as the major source of run terminations controlling translocation processivity. (**A**) Kinetic model of the RecQ ATPase cycle during ssDNA translocation, with rate constants representing best-fits or limit values determined from experimental data (lower bounds for *k*_1_, *k*_–2_, *k*_3_ and *k*_4_; upper bound for *k*_–3_; associative rates given for pseudo-first-order conditions) (Table 1). Also shown are the rate constants of RecQ dissociation from ssDNA in different nucleotide states (downward arrows; (11)), resulting in the specified run termination probabilities. Specified kinetic parameters and color-coding of enzymatic states apply to all panels. (**B**) Time course of the distribution of enzyme intermediates (main panel) and mechanochemical (P_i_-producing) cycles (inset) during a single processive run along quasi-infinite length ssDNA. (**C**) Upper bar: fractional abundance of RecQ enzymatic states within the ssDNA-bound population during translocation. Lower bar: distribution of run termination events occurring in different enzymatic states. (**D**) Effect of a 2-fold change (in either direction) in the DNA dissociation rate constant of individual RecQ enzymatic states on the processive run length of ssDNA translocation.

The presented results allowed the construction of a well-constrained kinetic model of the RecQ mechanoenzymatic cycle during ssDNA translocation (Figure 4A). This model reveals the distribution of DNA-bound enzymatic states, while being consistent with the macroscopic translocation parameters determined earlier (9,10,15). The model includes previously determined rate constants of dissociation of RecQ from ssDNA in different nucleotide states (11). Figure 4B shows the simulated time course of the distribution of enzyme intermediates during a single processive run of ssDNA translocation. (Note that single-run conditions are different from single ATP turnover conditions applied in Figures 2A and 3A.) The run is initiated by mixing the RecQ.ssDNA complex with excess ATP and is terminated by dissociation of RecQ from ssDNA dictated by the specified kinetic constants. During translocation, the majority (∼76%) of ssDNA-bound RecQ molecules populate the ATP-bound (prehydrolytic) state due to the rate-limiting ATP hydrolysis step (Figure 4B and C). The ADP-bound state is also significantly populated (to ∼15%), as ADP release is only about five times more rapid than ATP hydrolysis (Table 1). The different enzymatic states, however, exhibit widely varying DNA dissociation kinetics, with the ADP-bound state showing the most rapid ssDNA dissociation (Figure 4A) (11). This feature causes the majority (∼76%) of run terminations to occur in the ADP-bound state, despite the moderate steady-state abundance of this species (Figure 4C). Thus, the model implies that the strength of the DNA interaction of the RecQ.ADP state is an important determinant of translocation processivity. Accordingly, simulations imposing a moderate (2-fold) increase or decrease in the DNA dissociation rate constant of each individual enzymatic state revealed that the processive run length is markedly more sensitive to such changes in the RecQ.ADP state than in any other enzymatic species (Figure 4D).

We note that the simulations of Figure 4A and B predict a lower mean number of mechanochemical cycles during a translocation run (∼23; Figure 4B, inset) than that determined in our earlier study (100–320, Table 2; (9)). The difference likely arises from the condition that the longer translocation run lengths were determined for quasi-infinite length DNA substrates (M13 phage DNA, poly-dT; (9)), whereas the ssDNA interaction (11) and ATPase transient kinetic data (current study) were determined using dT_54_. However, the simulated run length of Figure 4B inset is consistent with corresponding values obtained for dT_54_ (9), and those determined by others under different conditions (10,15). The simulated run length is also influenced by *k*_4_ (ADP release rate constant), as this value controls the exit from the weakest ssDNA-interacting state (Figure 4A). Quenched-flow experiments using unmodified ATP yielded a higher *k*_4_ value than did mdADP experiments, indicating that the fluorescent label may slightly strengthen the RecQ–nucleotide interaction (Table 1). However, the main conclusions drawn from the simulations (greatest abundance of the ATP-bound state, run terminations controlled through the ADP-bound state) were robustly reproduced and unaffected by these variations.

**Table 2.** Mechanistic differences between RecQ homologs

	*E. coli* RecQ	Human BLM^a^
Rate-limiting step		
DNA-free ATPase	ATP hydrolysis (productive)^b^	Non-productive ATP hydrolysis^b^, transition between ADP-bound states
DNA-bound ATPase	ATP hydrolysis (productive)^b^	Transition between ADP-bound states

Kinetic enhancement by DNA^c^		
Steady-state ATPase	60–280	330–410
ATP binding (*k*_1_)	1	1
ATP hydrolysis (*k*_2_)	110	n.d.
Productivity of ATP hydrolysis (*k*_3_/*k*_–2_)^b^	0.7	>200
ADP release (*k*_4_)	1	14

Macroscopic parameters of ssDNA translocation		
*C* (ATP hydrolyzed/nt traveled)^d^	1.1 ± 0.2^g^	0.87 ± 0.08
*k*_trans_ (s^−1^)^e^	30–37	27–33
*k*_off,int_ (s^−1^)^f^	0.12^g^	0.2–0.6
Mean run length (nt)	100–320^g^	50

^a^Ref. (13).

^b^ATP hydrolysis is considered productive if the rate constant of P_i_ release (*k*_3_) exceeds the reverse rate constant of the hydrolysis step (*k*_–2_).

^c^Ratio of DNA-bound and DNA-free parameters. Nomenclature refers to Figure 1A.

^d^Mechanochemical coupling stoichiometry.

^e^ATP hydrolysis rate constant during ssDNA translocation.

^f^Net rate constant of enzyme dissociation from ssDNA during translocation.

^g^Parameters from (9), determined in conditions identical to those in the current study and (13).

Earlier we detected that, during ssDNA binding, RecQ helicase and its nucleotide complexes undergo an isomerization strengthening DNA interaction (11). The DNA dissociation rate constants in the simplified model of Figure 4A are rates of exit from the strongly DNA-bound (i.e. isomerized) form of each RecQ-nucleotide state. Simulations including all parameters of the two-step DNA interaction of RecQ produced results that were indistinguishable from those of Figure 4, as the weakly DNA-bound form was populated by only ∼1% of RecQ–DNA complexes during ssDNA translocation.

## DISCUSSION

### Energetic and structural implications for enzymatic tuning of DNA processing

Efficient coupling of the ATP-hydrolytic activity to translocation is generally associated with a marked DNA-induced allosteric activation of the ATPase activity of helicases (1). Here we show that, in *E. coli* RecQ, this activation is almost exclusively brought about by kinetic enhancement of the ATP cleavage step (Table 1). Nonetheless, all steps of the DNA-bound RecQ enzymatic cycle appear fairly reversible with the exception of P_i_ release, which therefore appears to be the thermodynamic driving force of the ATPase cycle, with energetic implications for ssDNA translocation. Earlier we proposed that RecQ complexed with the nucleotide analog ADP.AlF_4_ adopts a strongly DNA-bound, ‘clamped’ conformation (11). This state may be analogous to the post-hydrolytic RecQ.ADP.P_i_ complex, implying that P_i_ release is triggered by the formation of extensive enzyme–DNA interactions, probably involving the accessory DNA-binding (winged helix and HRDC) domains.

An intriguing implication of the model of Figure 4A is that translocation run terminations are largely controlled through the ADP-bound RecQ state. Even small changes in the DNA interaction (DNA dissociation rate constant) of RecQ.ADP will markedly influence run length, unlike in other nucleotide states (Figure 4D). Various structural features of complex physiological DNA substrates (e.g. the presence of a displaced DNA strand and/or branched structures in the vicinity of the enzyme) may significantly influence dissociation of RecQ from DNA. Thus, the proposed mechanism creates the possibility for precise tuning of the processivity of RecQ-catalyzed physiological reactions including invasion disruption, branch migration and/or nucleoprotein displacement. It remains to be determined how the helicase core (two tandem RecA-like domains) and the accessory DNA binding domains of RecQ contribute to DNA structure-dependent enzymatic tuning.

### Relation to other SF1–2 helicase mechanisms

The reversibility of nucleotide binding appears to be a common feature of SF1–2 helicases, which can be attributed to the two tandem RecA domains forming the ATP binding pocket of these enzymes (1,16). Nucleotide binding was observed to be a two-step process both in the absence and presence of DNA in Rep (17) and RecD2 helicases (18), whereas it occurred in a single step in the NA-free cycle and in two steps in the NA-bound cycle of PcrA (19), Ms116 (20) and DbpA (21) helicases. The second step of ATP binding was proposed to be a rate-limiting conformational change triggering ATP hydrolysis in RecD2 and PcrA helicases (18). Our data do not rule out that such an isomerization may occur also in DNA-bound RecQ helicase, facilitating tight mechanochemical coupling.

Similar to RecQ, rate-limiting ATP cleavage was proposed for RecG helicase (22). In DbpA the hydrolysis step appeared to be reversible and kinetically unfavorable in the absence of RNA, but activated by the NA strand (21).

As in RecQ, rapid and irreversible P_i_ release was proposed for PcrA (19), RecD2 (18) and RecG (22). Contrary, P_i_ release in DbpA contributes to the limitation of the RNA-activated steady-state ATPase activity (21).

### Mechanistic differences between RecQ homologs

Comparison of the mechanism of DNA activation of the ATPase cycles of *E. coli* RecQ and human BLM enzymes (13) reveals the following important differences between RecQ family members (Table 2). (i) The RecQ cycle is kinetically limited by ATP hydrolysis, whereas in BLM, a transition between two different ADP-bound states is rate-limiting. (ii) The DNA-induced enhancement of the ATPase activities of the two enzymes is of similar magnitude but brought about by different underlying mechanisms: acceleration of ATP hydrolysis in RecQ versus an increase in the productivity of ATP hydrolysis (i.e. the *k*_3_/*k*_–2_ ratio) plus the acceleration of the isomerization of the enzyme.ADP complex in BLM. Interestingly, the different underlying mechanisms of RecQ and BLM result in generally similar macroscopic features of translocation along simple ssDNA substrates (Table 2). It remains to be determined whether and how these differences in kinetic tuning determine the outcome of processing of complex multi-stranded DNA substrates.
